# Construction Notes

**Published:** 1905-10-14

**Authors:** 


### CONSTRUCTION NOTES.

A new workhouse infirmary has been opened at North Evington.

The committee of the Newcastle and Northumberland Branch of the National Association is appealing for funds to enable the sanatorium at Barrasford-on-Tyne to be completed and opened.

A new infirmary has been erected for the Bury Union. There is accommodation for 126 patients now, and it is intended to add 75 beds later. The cost was £21,000. The architect is Mr. H. Hopkinson, M.I.C.E.

The Public Health Committee of the Derbyshire County Council called last week a conference to discuss a proposal to provide a sanatorium for consumption for the county, containing forty beds. The delegates of the Urban and Rural Councils undertook to recommend their authorities to support the suggestion.

The Bristol Royal Infirmary has had a very successful year. No doubt interest was stimulated by the Carnival. It is remarkable that no less than £1,159 was collected in the boxes in the tramcars.

A new dispensary at Hyson Green has been erected for Nottingham. It contains waiting halls, dressing, accident and surgical rooms, besides the usual offices, and a dispensing department. A dwelling is provided for the surgeon. The cost has been £3,000. The architect was Mr. E. Sutton, of Nottingham.

The Manchester Infirmary has recently received a donation of £2,500 from the Misses Barnes, of Wilmslow, in memory of the late Vicar of Lindow. Of this sum £1,500 is to be devoted to the endowment of a memorial bed. The infirmary will also receive £5,000 through the will of the late Mr. John Harling.

